# Dynamic Transcriptome Reveals the Mechanism of Liver Injury Caused by DHAV-3 Infection in Pekin Duck

**DOI:** 10.3389/fimmu.2020.568565

**Published:** 2020-11-06

**Authors:** Junting Cao, Yunsheng Zhang, Ying Chen, Suyun Liang, Dapeng Liu, Wenlei Fan, Yaxi Xu, Hehe Liu, Zhengkui Zhou, Xiaolin Liu, Shuisheng Hou

**Affiliations:** ^1^ Shaanxi Key Laboratory of Molecular Biology for Agriculture, College of Animal Science and Technology, Northwest A&F University, Yangling, China; ^2^ Ministry of Agriculture Key Laboratory of Animal Genetics Breeding and Reproduction (Poultry), Institute of Animal Science, Chinese Academy of Agricultural Sciences, Beijing, China; ^3^ College of Food Science and Engineering, Qingdao Agricultural University, Qingdao, China

**Keywords:** duck hepatitis A virus 3, RNA-seq, duck, liver, apoptosis

## Abstract

Duck hepatitis A virus 3 (DHAV-3) is a wild endemic virus, which seriously endangers the duck industry in China. The present study aims to elucidate the mechanism of duck resistance to DHAV-3 infection. Both resistant and susceptible ducks were challenged with DHAV-3 in this experiment. The histopathological features and serum biochemical indices (ALT and AST) were analyzed to estimate liver injury status at 6, 12, 15, and 24 h post-infection (hpi). The dynamic transcriptomes of liver were analyzed to explain the molecular regulation mechanism in ducks against DHAV-3. The result showed that the liver injury in susceptible ducks was more serious than that in the resistant ducks throughout the four time points. A total of 2,127 differentially expressed genes (DEGs) were identified by comparing the transcriptome of the two populations. The expression levels of genes involved in innate immune response increased rapidly in susceptible ducks from 12 hpi. Similarly, the expression of genes involved in cytokine regulation also increased at the same time points, while the expression levels of these genes in resistant ducks remained similar between the various time points. KEGG enrichment analysis of the DEGs revealed that the genes involved in cytokine regulation and apoptosis were highly expressed in susceptible ducks than that in resistant ducks, suggesting that excessive cytokine storm and apoptosis may partially explain the mechanism of liver injury caused by DHAV-3 infection. Besides, we found that the *FUT9* gene may contribute to resistance towards DHAV-3 in resistant ducklings. These findings will provide insight into duck resistance and susceptibility to DHAV-3 infection in the early phases, facilitate the development of a strategy for DHAV-3 prevention and treatment, and enhance genetic resistance *via* genetic selection in animal breeding.

## Introduction

Duck hepatitis A virus 3 (DHAV-3) is a threat to the duck industry in China (1). For ducklings aged below 14 days, the mortality rate caused by DHAV-3 infection will reach almost 90% (2). DHAV-3 is characterized by its rapid transmission. Opisthotonos posturing and severe liver multiple ecchymoses occur in dead ducklings. There are three serotypes of DHAV, DHAV-3 is more epidemic in China than the other two serotypes (DHAV-1, -2) (3–6). According to the epidemiological investigations, DHAV-3 strain has been the predominant viral type in China since 2013, with high morbidity and mortality, causing economic loss to the intensive duck production farms (7, 8). DHAV-3 has become one of the major pathogens harming the development of the duck industry.

Traditionally, vaccines used for DHAV prevention are mainly effective in DHAV-1 infection, with no effect against heterologous viruses (9–11). As there are no effective DHAV-3 prevention measures, genetic selection of birds is considered an efficient and permanent way to control DHAV-3 infection. Based in part on high mortality rates, selective breeding has led to the development of lines of chickens, which display resistant or susceptible phenotypes (12–19). Similarly, we selected ducklings with resistant or susceptible phenotypes. Recently, RNA-seq technology has been widely used in virus-animal/plant interactions (20–23). Analysis of host mRNA expression after a challenge provides insight into the host-pathogen interaction. Understanding the host response to DHAV-3 is necessary to develop improved solutions to combat this disease. A previous study performed RNA-seq analysis of the feet and lymph nodes at peak viraemia, acute arthritis and chronic disease in the CHIKV adult wild-type mouse model and found out that granzyme A is an essential driver of arthritic inflammation (22). Therefore, we hypothesized that the genes encoding immune system molecules are differentially expressed in susceptible and resistant ducklings following experimental infection with DHAV-3. Furthermore, we intended to elucidate these differences in immune system gene expression patterns in different ducklings that display DHAV-3 resistant and susceptible phenotypes at 6, 12, 15, and 24 h post-infection (hpi), which coincides with the dynamic changes in injury post-DHAV-3 infection. In this experiment, we used selected ducks with different susceptibility to compare their dynamic responses against DHAV-3 in the liver. A comparison of liver transcriptome will explain the different immune responses in ducklings after DHAV-3 infection. The RNA-seq analysis will provide us with several genes that may be key players involved in regulating the differences in pathogenesis and host defense mechanisms post DHAV-3 infection.

Our study will present a comprehensive characterization of the liver in DHAV-3 infected ducklings, and it will provide insights into the pathogenic mechanism in ducks infected with DHAV-3.

## Materials and Methods

### Animals and Viruses

Resistant and susceptible Pekin ducklings were obtained from the Pekin Duck Breeding Center (Chinese Academy of Agricultural Sciences, Beijing, China). The experimental ducklings’ parent stocks were tested for DHAV-3 antibody-free status using a Duck DHAV-3 ELISA kit (Biorbyt, Shanghai, China).The virus used in this study was the 112803 strain of DHAV-3, as used in previous study (2). The viral titer was 10^5.83^ 50% egg lethal dose (ELD_50_) per 0.2 mL.

### Experimental Procedures

Two generations of the resistant and susceptible ducklings were selected in our lab. As for the susceptible ducks, we collected the full sibling ducklings with mortality of more than 70% after challenging with DHAV-3, and ducks with mortality less than 10% were regarded as the resistant ducks. All the ducks were challenged with DHAV-3 at 7 days of age. For the susceptible and resistant ducklings, we selected the full sibling ducks. The susceptible and resistant ducks were selected from the previous generation with the same standard. The breeding plan is consistent with the method described in previous study in our lab. In this study, we still use the selecting method.

Pilot and formal experiments were conducted of this generation in this study. The pilot experiment was conducted to select resistant and susceptible experimental ducklings for use in the formal experiments. A total of 554 3-day-old ducklings were selected in the pilot experiment. In this study, we define the ducklings with mortality rate of more than 70% as the susceptible group (S), while those with a mortality rate lower than 10% were regarded as the resistant group (R). All the ducklings received ad libitum access to water and feed. Twenty-four hours of light were provided daily.

Three livers were collected from each group at 6, 12, 15, and 24 hpi. The right lobes of the liver specimens were immediately cryopreserved in liquid nitrogen until RNA isolation was performed. Blood samples were collected into tubes without anticoagulant. Sera were obtained after centrifuging at 1,520 × *g* for 15 min. Additionally, the liver specimens were fixed in a 4% paraformaldehyde solution for histopathological examination.

### Serum Biochemistry Analysis

Sera were stored at −20°C for subsequent experiments. The serum content of alanine aminotransferase (ALT) and aspartate aminotransferase (AST) (Maccura, Sichuan, China) were analyzed using a biochemical analyzer (Hitachi 7080 Automate, Tokyo, Japan) according to the manufacturer’s instructions.

### Hematoxylin and Eosin (H & E) Staining

The liver samples fixed in 4% paraformaldehyde solution were dehydrated, embedded in paraffin, cut into 4-μm-thick sections, and stained with H & E (Solarbio, Beijing, China) using standard procedures according to the manufacturer’s instructions.

### RNA Extraction, Quality Analysis, Library Preparation, and RNA Sequencing

Total RNA was extracted from 50 mg frozen liver tissue using Trizol reagent (Takara, Dalian, China) according to the manufacturer’s instructions. A Nanodrop 2000 system (Thermo Fisher Scientific, Illkirch, France) was used to preliminary quantify the RNA content, and Agilent 2100 RNA 6000 Nano kit was used for precise quantification. The mRNA was enriched from total RNA using oligo (dT) magnetic beads, and cDNA was synthesized from reverse transcription using a random hexamer-primer. A total of 24 cDNA libraries were produced for RNA-seq, with three susceptible and three resistant ducklings per time point. The Illumina NovaSeq 6000 sequencing platform was used for RNA sequencing in this study.

### Analysis of Differently Expressed Genes

EdgeR software was used to identify the differentially expressed genes (DEGs) in diﬀerent infected livers. As there were two groups of ducklings and four time points in this study, we performed differential gene expression analyses using two strategies.

An initial data filtering step was used to remove sequencing adaptors and low-complexity reads. Read count values of each transcript were calculated using TopHat software. The paired-end reads were mapped against the Pekin duck reference genome. Counts per million (CPM) mapped sequence read values were calculated and the DEGs were identified using the edgeR software package. A *P*-value < 0.05 and |log_2_ fold change| > 2 were set as the thresholds for significant differential expression.

### Function Enrichment Analysis of the DEGs

The KEGG pathway analysis of DEGs was enriched to further define DEG function in duckling livers post DHAV-3 infection. The enrichment KEGG pathways were selected according to a *P*-value < 0.05. Protein sequences were obtained for all the DEGs. The online website KOBAS (http://kobas.cbi.pku.edu.cn/index.php) was used to perform the KEGG enrichment. The species, which was chosen to perform the KEGG analysis was *Gallus gallus*.

### Apoptosis Verification Using TUNEL Assay

Liver samples fixed in 4% paraformaldehyde were used to perform terminal dUTP nick end labeling (TUNEL) using an apoptosis detection kit according to the manufacturer’s instructions (Beyotime, Shanghai, China). All cells, including apoptotic cells, were observed under a fluorescence microscope. The TUNEL-positive cells were quantified by randomly counting three different microscopic fields for each section. Image-Pro Plus 6.0 software was used for picture analysis. Ratio of apoptotic cells was calculated as the number of TUNEL-positive nuclei divided by the total number of nuclei.

### Statistical Analysis

The data were expressed as the means ± SD from at least three independent experiments. The t-test was performed to compare the difference using SAS 9.4.

## Results

### Gross Lesions and Histopathological Analysis

**Figure 1:**
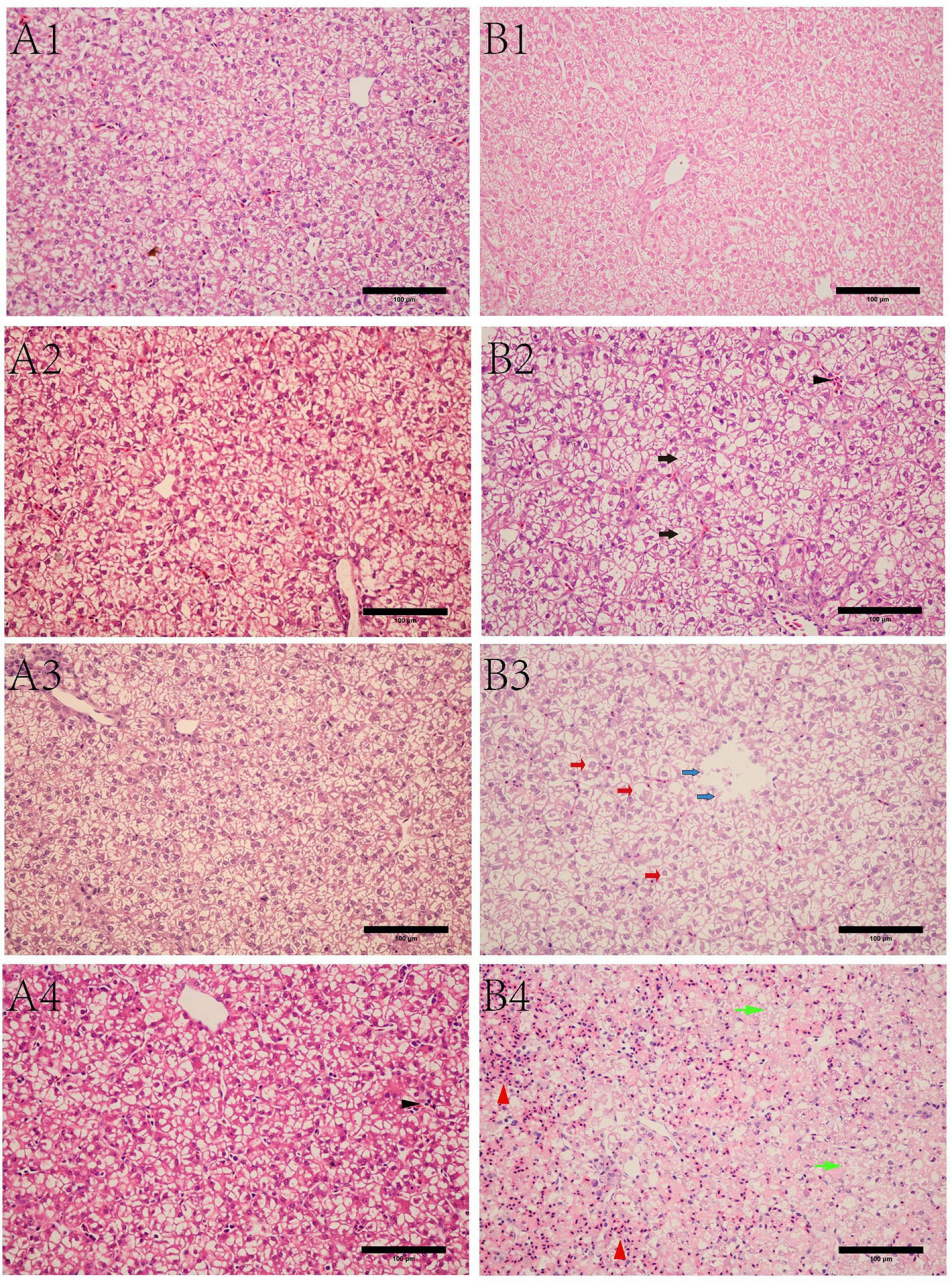
Microscopic lesions of livers in different ducks at different time points post infection. **(A)** resistant group. A1, A2, A3, A4 refer to liver sections from the resistant group at 6, 12, 15, 24 hpi, respectively. **(B)** susceptible group. B1, B2, B3, B4 refer to liver sections from the susceptible group at 6, 12, 15, 24 hpi, respectively. Black arrow in B2 refers to granule denaturation, while black triangle is the inflammatory cell infiltration. Red arrow in B3 refers to ballooning degeneration, while blue arrow refers to necrolysis. Green arrow in B4 refers to steatosis, while the red triangle refers to hemorrhage. Bar = 100 μm.

Livers were collected at 6, 12, 15, and 24 hpi, and we found that the gross lesions in the susceptible ducklings underwent dynamic changes and peaked at 24 hpi (**Figures 1B1–B4**). Under light microscope, the liver in the resistant ducklings showed a normal structure with wellarranged hepatocyte cords (**Figures 1A1–A4**. We observed ecchymoses hemorrhage in the livers of susceptible ducklings at 15 hpi (**Figure 1B3**), hemorrhage got even worse at 24 hpi. At 12 hpi, granular degeneration was detected in the liver sections, as well as that at 15 hpi (**Figures 1B2, B3**). We observed infiltration of the hepatic sinusoids by large numbers of red blood cells, which was accompanied by disordered hepatic cords in the livers of susceptible ducklings at 24 hpi, exhibiting obvious hemorrhage and swelling (**Figure 1B4**). Additionally, parts of the cell nuclei underwent pyknosis, karyolysis, or karyorrhexis.

### Detection of Biochemical Markers in Serum Samples

**Figure 2:**
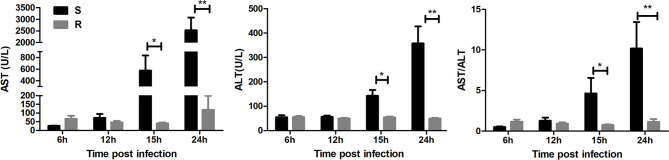
Detection of serum biochemical AST, ALT, AST/ALT. *P < 0.05, **P < 0.01.

Serum biochemical markers (ALT and AST) were measured to estimate the degree of hepatic injury in susceptible and resistant ducklings post DHAV-3 infection. As shown in **Figure 2**, for the two markers, high levels were detected at 15 and 24 hpi in susceptible ducklings, which were significantly higher than those of resistant ducklings. Besides, we calculated the ALT/AST level (**Figure 2**), which indicated the degree of hepatic injury, and the result was consistent with the ALT and AST.

### Apoptosis Analysis

**Figure 3:**
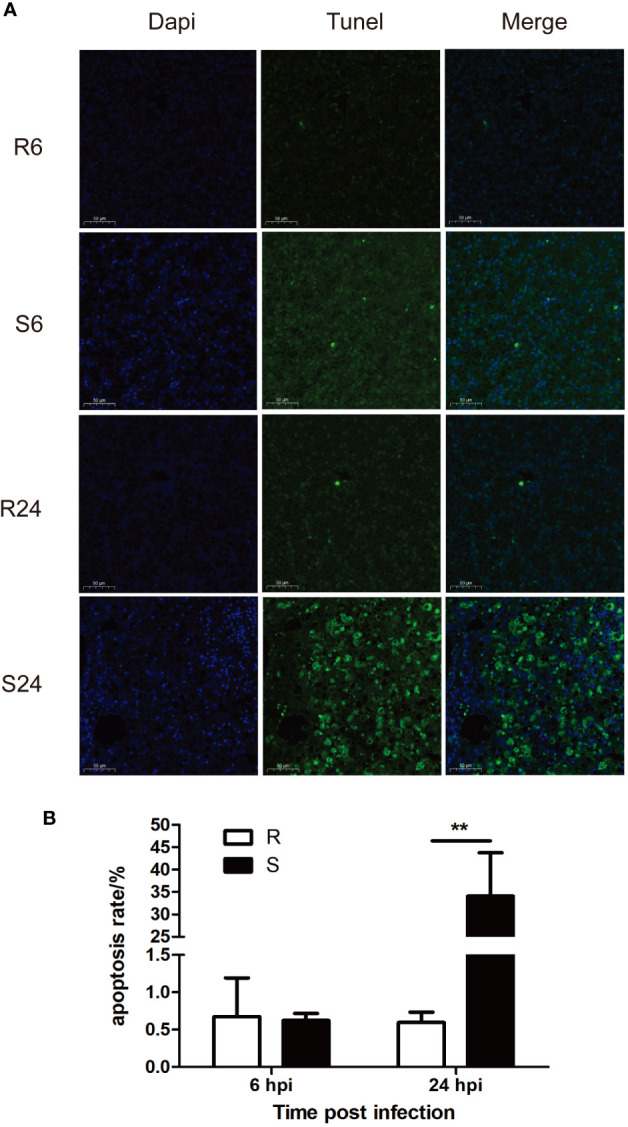
Effect of DHAV-3 infection on liver cell apoptosis. **(A)** TUNEL staining results. **(B)** statistical analysis of apoptosis rate. Data were presented as means ± SD. R6 and R24 refer to the liver TUNEL staining at 6 and 24 hpi in the resistant ducklings, while S6 and S24 refer to the liver TUNEL staining at 6 and 24 hpi in the susceptible ducklings **P < 0.01.

Obvious differences in apoptosis were observed between the resistant and susceptible groups at 24 hpi. A large number of apoptotic cells was seen in liver tissue in the susceptible group at 24 hpi. There was no difference between the two groups at 6 hpi (**Figure 3**). A significant difference in apoptosis was detected in the two groups. At 24 hpi, the susceptible group showed a significant increase in the number of apoptotic cells (*P* < 0.05) (**Figure 3B**).

### Transcriptional Profiling and Different Gene Expression Analysis

**Figure 4:**
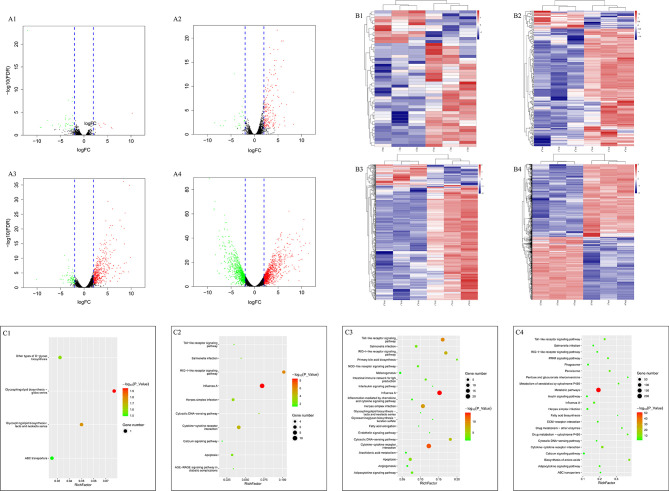
Volcano, heatmap and KEGG pathway analysis of DEGs between the two groups. **(A–C)** refer to volcano, heatmap and KEGG enrichment pathway, respectively. A1, B1 and C1 refer to the comparation between the two groups at 6 hpi, A2, B2, and C2 refer to the comparation between the two groups at 12 hpi, A3, B3, and C3 refer to the comparation between the two groups at 15 hpi, A4, B4, and C4 refer to the comparation between the two groups at 24 hpi. While in the volcano map, the red points refer to the up-regulated genes and the green points refer to the down-regulated genes. The results were obtained through susceptible ducklings versus resistant ducklings.

A total of 11,683 genes were obtained in this study. Firstly, we compared the DEGs between the two groups at the same time points. We observed 46, 205, 545, and 1,954 genes differently expressed at 6, 12, 15, and 24 hpi, respectively (**Table 1**). The up- and down-regulated genes are shown in the volcano map (**Figures 4A1–A4**) and heat map (**Figures 4B1–B4**).

**Table 1:** Numbers of DEGs in different comparation.

Number	Up	Down	Total
6	11	35	46
12	178	27	205
15	461	84	545
24	1,051	903	1,954
R6-12	20	27	47
R6-15	56	24	80
R6-24	42	42	84
S6-12	313	31	344
S6-15	660	73	733
S6-24	1,177	863	2,040

Note: 6, 12, 15, 24 refer to the DEGs obtained through comparation between the two groups at 6, 12, 15, 24 hpi, respectively. The results were obtained through susceptible ducklings versus resistant ducklings. While R6-12, R6-15, R6-24 respectively refers to the DEGs numbers of 12, 15, 24 hpi compared to 6 hpi in resistant ducklings, S6-12, S6-15, S6-24 refers to the DEGs numbers of 12, 15, 24 hpi compared to 6 hpi in susceptible ducklings, respectively.

**Figure 5:**
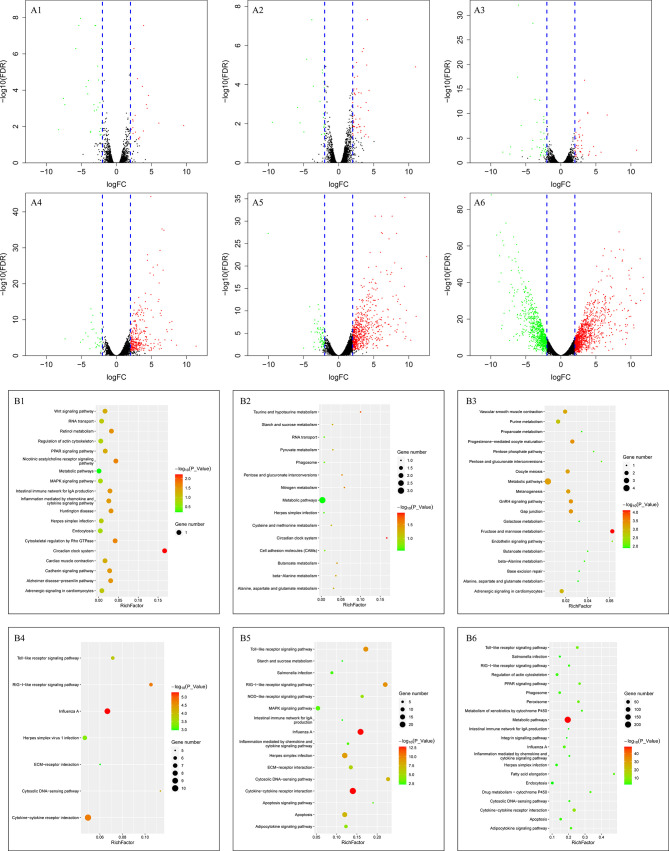
Volcano and KEGG pathway analysis of DEGs in one group. **(A, B)** refer to volcano and KEGG enrichment pathway, respectively. A1, A2, and A3 showed the DEGs analysis of 12, 15, and 24 hpi compared to 6 hpi in resistant ducklings, respectively, while A4, A5, and A6 showed that in susceptible ducklings. B1, B2, and B3 showed the KEGG analysis of DEGs obtained from 12, 15, and 24 hpi compared to 6 hpi in resistant ducklings, respectively, while B4, B5, and B6 showed that in the susceptible ducklings (*P*-value < 0.05 and |log_2_ fold change| > 2).

In addition, the gene expression data of 12, 15, 24 hpi were compared to the data of 6 hpi in each group. For the resistant group, 47, 80, 84 DEGs were observed. But 344, 733, and 2,040 DEGs were detected in the susceptible group (**Table 1**). The numbers of DEGs can be seen in **Table 1** and the volcano maps show the number of all genes, including the up- and down-regulated genes (**Figures 5A1–A6**).

### Pathway Analysis of DEGs Based on KEGG After DHAV-3 Infection

For pathways of DEGs at the same time points in the two groups, the phase was divided into four parts according to the results. The pathways indicated different changes between the two groups. At 6 hpi, the pathway enriched was the glycosphingolipid biosynthesis pathway (**Figure 4C1**), and the DEGs enriched in the pathway was *FUT9*. At 12 hpi, the innate immune system seemed to have been activated. Influenza A, RIG-I-like receptor signaling pathway, herpes simplex infection, and salmonella infection were enriched at 12 hpi (**Figure 4C2**). Besides, some DEGs were enriched in inflammation mediated by chemokine and cytokine signaling pathways. At 15 hpi, different functional categories were enriched, including innate immune pathways, metabolism pathways, angiogenesis, and so on (**Figure 4C3**). At 24 hpi, except the innate immune pathways, metabolic pathways, biosynthesis of amino acids, and metabolism of amino acids were enriched (**Figure 4C4**).

For the resistant ducklings, compared to the 6 hpi, the DEGs were enriched in the intestinal immune network for IgA production, the only pathway which was related to immunity at 12 hpi (**Figure 5B1**). At 15 and 24 hpi, no immune pathway was enriched (**Figures 5B2, B3**). For the susceptible ducklings, compared to 6 hpi, KEGG pathway enrichment analyses revealed enrichment for terms including Influenza A, RIG-I-like receptor signaling pathway, cytosolic DNA-sensing pathway, cytokine-cytokine receptor interaction, toll-like receptor signaling pathway, herpes simplex virus 1 infection, ECM-receptor interaction, death receptor signaling, signal transduction, TRAF-mediated activation of IRF, cadherin signaling pathway, inflammation mediated by chemokine and cytokine signaling pathway, and apoptosis (**Figures 5B4–B6**). The different enriched pathways indicate the dynamic changes that occurred in the liver. KEGG pathway enrichment indicated that multiple vital biological processes are involved in DHAV-3 infection in ducklings (**Figures 5B1–B6**).

### Several Genes Were Upregulated in Susceptible Ducklings but Not in Resistant Ducklings After Infection

**Figure 6:**
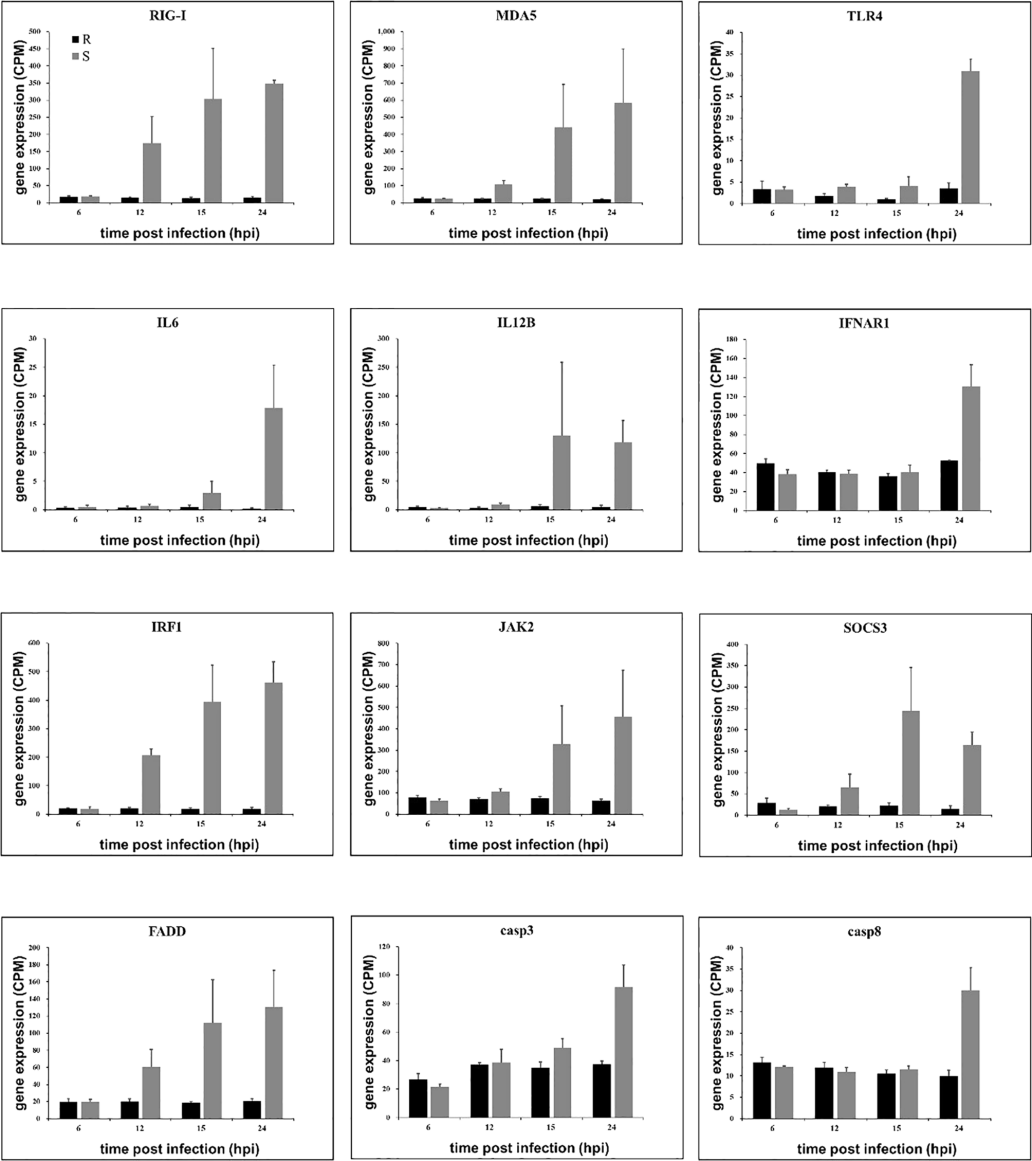
Gene expression of related genes. Expression levels (Counts per million, CPM) of genes as determined from RNA-seq are presented as averaged CPM at each time point (means ± SD, n = 3). The selection standard for DEGs is *P* value < 0.05 and |log_2_ fold change| > 2.

Similarly, the host response in susceptible ducklings is characterized by a stronger inflammatory response. Therefore, we selected several genes that were related to inflammatory-induced. We found these genes were upregulated in the susceptible ducklings (**Figure 6**). As the high expression of these genes in susceptible ducklings, there may be some connection between the gene expression and the liver damage in susceptible ducklings.

DEGs with different functional categories that were upregulated in susceptible ducklings were identified. Genes such as pattern recognition receptors retinoic acid-inducible gene-I (*RIG-1*), melanoma differentiation-associated gene 5 (*MDA5*), toll-like receptor 4 (*TLR4*), inflammatory cytokines interleukin 6 (*IL6*), interleukin 12B(*IL12B*), interferon alpha and beta receptor subunit 1 (*IFNAR1*), inflammatory regulator interferon regulatory factor 1 (*IRF1*), Janus kinase 2 (*JAK2*), suppressor of cytokine signaling 3 (*SOCS3*), and the apoptosis-associated protein fas associated *via* death domain (*FADD*), *caspase 3*, and *caspase 8* were detected to be upregulated in susceptible ducklings, which may explain the injury that occurred in the susceptible ducklings.

### Expression of DEGs Related to Resistance and Susceptibility

**Figure 7:**
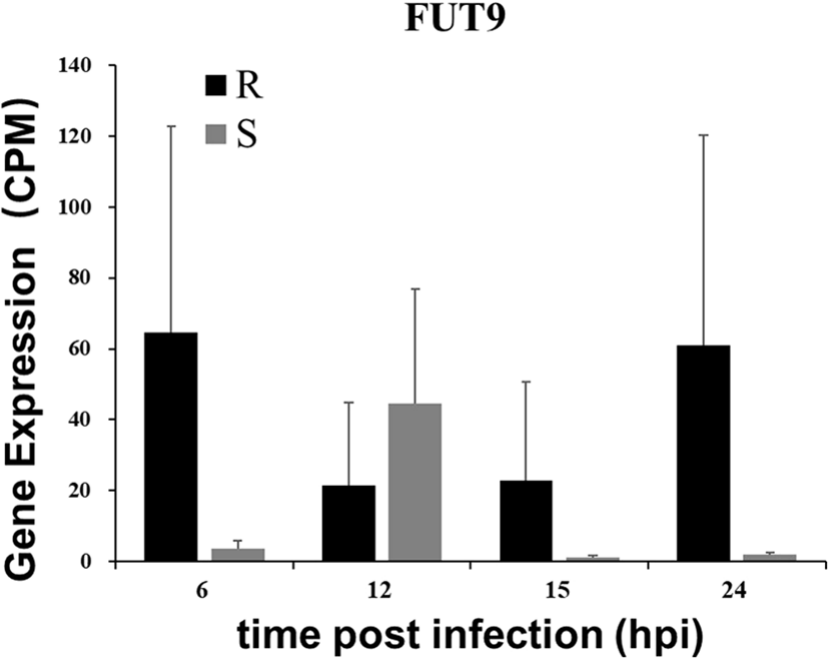
Gene expression of *FUT9.* Expression levels of *FUT9* as determined from RNA-seq are presented as averaged CPM at each time point (means ± SD, n = 3). The selection standard for DEGs is *P* value < 0.05 and |log_2_ fold change| > 2.

Several genes were upregulated in resistant ducklings but not in susceptible ducklings after infection. These genes may play roles in resistance towards DHAV-3 in resistant ducklings. Firstly, we performed an analysis through edgeR software for all genes in all groups to identify genes that were significantly upregulated (False Discovery Rate, FDR < 0.05). From this set, genes that were upregulated in resistant ducklings were selected. Two DEGs were significantly upregulated at 6, 15, and 24 hpi in resistant ducklings: *FUT9* and *LOC110352387* (**Figure 7**). The low expression level of *LOC110352387* makes it an unlikely crucial candidate mediating the difference in susceptibility between resistant and susceptible ducklings.

## Discussion

Herein, we performed RNA-seq based analysis of gene expression changes in a duckling DHAV-3 infection model by comparing a resistant duckling group, resistant duckling that survives DHAV-3 infection with a highly susceptible group, for which infection with DHAV-3 is lethal. At 6, 12, 15, and 24 hpi, liver damage and apoptosis were observed, at the same time, 46, 205, 545, and 1,954 DEGs were obtained through RNA-seq analysis. In this study, the dynamic changes in the liver and gene expression profiles were combined to reveal the internal mechanism of liver injury and select candidate genes to explain the resistance towards DHAV-3 in resistant ducklings.

From the results of the liver section and serum biochemical markers, we found that injury only occurred in the susceptible ducklings. The ALT and AST levels increased at 15 hpi and peaked at 24 hpi. The sections of livers in susceptible ducklings showed injuries at 15 and 24 hpi; the hepatic sinusoids were infiltrated by large numbers of red blood cells, which were accompanied by serious fatty degeneration of hepatocytes.

ALT and AST are regarded as critical indicators in the diagnosis and assessment of liver disease (24, 25). ALT functions in the liver and is present there in abundance. The activity of ALT in the normal liver is about 3,000 times that of serum activity, while AST mainly exists in the mitochondrion of hepatocytes (24). Injury to hepatocytes causes ALT and AST in the hepatocytes to be released into the serum. Notably, damage to the hepatocytes was observed at 15 hpi. A previous study found that there were positive correlations between AST/ALT levels and the degree of lobular inflammation (26), which is consistent with our results. The liver sections showed that inflammatory cell inﬁltration occurred at 15 and 24 hpi. All the results indicate that damage to the liver occurred at 15 and 24 hpi.

DHAV-3 is a single-stranded RNA virus, which belongs to the *Picornaviridae* family. Pathogen-associated molecular patterns (PAMPs) are essential for the life-cycle of the pathogen, which initiates the host-pathogen interactions (27, 28). Multiple pattern recognition receptors (PRRs) exist in macrophages, dendritic cells, and various nonprofessional immune cells, including toll-like receptors (TLRs), RIG-I-like receptors (RLRs), NOD-like receptors (NLRs), and C-type lectin receptors (CLRs) (29). The PRRs could sense the virus immediately through PAMPs and activate the innate immune response (30). Effective sensing of PAMPs rapidly induces host immune responses *via* the activation of complex signaling pathways that culminate in the induction of inflammatory responses mediated by various cytokines and chemokines, which subsequently facilitate the eradication of the pathogen (31).

In this study, we found several PRRs, which were up-regulated in the susceptible ducklings, such as *RIG-I*, *MDA5*, *TLR4*, *TLR5*, and *TLR7*. Toll-like receptor signaling pathway and RIG-I receptor signaling pathway can both trigger the innate immune response and promote the release of cytokines (32). In the RIG-I receptor signaling pathway, once the PRRs (*RIG-I* and *MDA5*) sense viral RNA, downstream molecules (TNFRSF1A associated *via* death domain (*TRADD*), *FADD*, TNF receptor associated factor 3 (*TRAF3*), and TANK binding kinase 1 (*TBK1*)) are activated. *TBK1* directly phosphorylates interferon regulatory factor 3 (*IRF3*) and interferon regulatory factor 7 (*IRF7)* promoting their dimerization and translocation into the nucleus. TRADD protein is a death domain protein containing an adaptor molecule that interacts with tumor necrosis factor receptor superfamily member 1A (*TNFRSF1A*/*TNFR1*) and mediates programmed cell death signaling and NF-kappa B activation. The downstream gene expression (*FADD*, *caspse3*, and *caspase8*) certified the apoptosis, which occurred in the susceptible ducklings. *TRADD* also contributes to the toll-like receptor signaling pathway, which resulted in the release of inflammation factors.

We also found that the Janus kinase/signal transducers and activators of transcription (JAK/STAT) signaling pathway was over activated in the susceptible ducklings. The expression of *STAT1*, *JAK1*, and *JAK2* genes increased following the infection time.

Canonical JAK-STAT signaling begins with the association between cytokines and their corresponding transmembrane receptors (33). Receptor oligomerization then precipitates the trans-activation of JAKs, which in turn, phosphorylates the cytoplasmic tails of the receptors to create the requisite docking sites for STATs. This puts JAKs and STATs in spatial proximity and allows the former to mediate tyrosine-phosphorylation (p-Tyr) of the latter, which results in STAT dimerization, nuclear translocation, DNA binding, and ultimately the modulation of gene transcription (34). In this study, overexpression of the genes in JAK-STAT signaling resulted in the over-release of cytokines, which led to inflammation in the liver.

Several processes occurred in the liver of the susceptible ducklings to avoid damage caused by inflammation. Signaling *via* the JAK-STAT signaling pathway is a dynamic process and it involves the rapid transmission of a signal from the cell membrane to the nucleus. Following the activation of the JAK-STAT signaling pathway was a highly organized response. The SOCS proteins are the primary drivers of signal attenuation, they are induced by cytokine exposure (via STAT) and then act as negative-feedback inhibitors to switch off the signaling cascade (33). Suppressor of cytokine signaling 3 (*SOCS3*) protein is described as a negative regulator of cytokine signaling mediated by the JAK-STAT signaling pathway. SOCS3 can inhibit JAK tyrosine kinase activity directly through its kinase inhibitory region (35). The deletion of *SOCS3* in mouse hematopoietic resulted in a severe inflammatory disease during adult life, which indicates that the *SOCS3* plays an important role in regulating the production of cytokines (36). Besides, protein inhibitor of activated STAT (*PIAS)* is also described as a negative regulator of the JAK-STAT signaling pathway (37). In this study, the expression level of *PIAS* increased at 15 and 24 hpi compared to previous time points. *PIAS* has been reported to negatively regulate *STAT* by inhibiting its translocation and phosphorylation. In this study, the induced expression of cytokine regulating genes (e.g. *SOCS3*) could therefore reflect the sustained secretion of proinflammatory cytokines in susceptible ducklings.

Persistent inflammation also exacerbates cell stress and tissue damage, causing cell apoptosis. Apoptosis was detected using TUNEL assay. In the susceptible ducklings, the apoptosis rate was significantly higher than that in the resistant group. Previous studies found that duck hepatitis A virus type 1 (DHAV-1) caused apoptosis in the liver after infection (38–40). In agreement with previous studies, the DHAV-1 infection drove apoptosis in the liver and the inflammatory response to viral infection could have exacerbated the level of apoptosis. For example, the level of the classical proinflammatory cytokine TNF-a has been reported to be associated with apoptosis (41). TNF superfamily member 10 (*TNFSF10*), also known as *TRAIL*, induces the signal transduction of apoptosis in tumor cells. It was shown recently that T cells can kill target cells *via TRAIL*/TRAIL receptor interaction, suggesting that *TRAIL* might serve as a cytotoxic effector molecule in activated T cells *in vivo*. Moreover, a previous study found that the HCV core protein can enhance TRAIL-mediated apoptotic cell death (42). In this study, the expression of the *TRAIL* gene increased during infection in susceptible ducklings, and the apoptosis rate at 24 hpi was significantly higher than that at 6 hpi. These results indicated that there was an association between apoptosis and *TRAIL* in DHAV-3 infection in susceptible ducklings.

Previous reports have shown that caspase-3 cleavage is one of the most important hallmarks of apoptosis activation in different models. A previous study found that both HBV and HCV infection induce caspase-3 cleavage in infected hepatocytes (43). In this study, *caspase 3* showed a significant upregulation at 24 hpi in the susceptible ducklings and the TUNEL assay verified the presence of apoptosis at this time point.

Interestingly, we observed that expression of *FUT9* was upregulated in the livers of resistant ducklings. The *FUT9* gene product belongs to the glycosyltransferase family. The protein catalyzes the last step in the biosynthesis of the Lewis X (LeX) antigen (44–47). Studies on the function of *FUT9* showed that Fut9^-/-^ mice, which were unable to synthesize the LeX structure, showed inhibition in inflammation (48). Clinically, the treatment of patients infected with highly virulent viruses during the early phase of infection includes the control of excessive innate immune responses. Besides, a single nucleotide polymorphism (SNP) located in the gene *FUT9*, rs3811070, was reported to be significantly associated with placental malaria infection (46). The high level of *FUT9* expression may play an important role in the early phase of DHAV-3 infection in resistant ducklings.

To summarize the above results, we can conclude that the cause of death in the susceptible ducklings was due to fatty degeneration, cytokine storm, and apoptosis in the liver, and the execution of liver function was destroyed, eventually leading to the death of the susceptible ducklings. For the resistant gene, the candidate gene *FUT9* may be important for host defense.

## Data Availability Statement

The datasets presented in this study can be found in online repositories. The names of the repository/repositories and accession number(s) can be found below: https://www.ncbi.nlm.nih.gov/, PRJNA609437 https://bigd.big.ac.cn/gsub/, PRJCA002148.

## Ethics Statement

The animal study was reviewed and approved by Committee of Animal Experiments of the Institute of Animal Sciences, Chinese Academy of Agricultural Sciences (IAS2018-13).

## Author Contributions

SH and XL conceived and coordinated the study. JC performed the study and wrote the manuscript. JC, YZ, YC carried out the bioinformatics and experiment analysis. SL, DL, WF, YX, HL and ZZ gave advices about concept and revised manuscript. All authors contributed to the article and approved the submitted version.

## Funding

This work was supported by grants from the National Natural Science Foundation of China (31772592), Chinese Agriculture Research System of Waterfowl (CARS-42) and Central Public-interest Scientific Institution Basal Research Fund (2019-YWF-YB-08).

## Conflict of Interest

The authors declare that the research was conducted in the absence of any commercial or financial relationships that could be construed as a potential conflict of interest.

## Supplementary Material

DEGs obtained through RNA-seq analysis were listed in **Table S1** in **Supplementary Material (S1)**. In the file, R6 vs S6, R12 vs S12, R15 vs S15, R24 vs S24 refer to the DEGs obtained from different groups at the same time point. R6 vs R12, R6 vs R15 and R6 vs R24 refer to DEGs of 12, 15, and 24 hpi compared to 6 hpi in resistant ducklings, respectively. And S6 vs S12, S6 vs S15 and S6 vs S24 refer to DEGs of 12, 15, and 24 hpi compared to 6 hpi in the susceptible ducklings, respectively.
